# Validity and reliability of the Critical-Care Pain Observation Tool (CPOT) for critically ill pediatric patients

**DOI:** 10.1371/journal.pone.0320373

**Published:** 2025-04-18

**Authors:** Haruhiko Hoshino, Mitsuki Ikeda, Yujiro Matsuishi, Yuki Enomoto, Nobutake Shimojo, Misaki Kotani, Shunsuke Kobayashi, Takahiro Kido, Satomi Hayashi, Yoko Furuya, Yoshiaki Inoue

**Affiliations:** 1 Adult Nursing, Department of Nursing, Faculty of Medical Technology, Teikyo University, Tokyo, Japan; 2 Department of Emergency and Critical Care Medicine, Faculty of Medicine, University of Tsukuba, Tsukuba, Ibaraki, Japan; 3 Adult and Elderly Nursing, Faculty of Nursing, Tokyo University of Information Science, Chiba, Japan; 4 Department of Pediatrics, University of Tsukuba Hospital, Tsukuba, Ibaraki, Japan; Stanford University School of Medicine, UNITED STATES OF AMERICA

## Abstract

**Introduction:**

In some regions, critically ill pediatric and adult patients are cared for in the same intensive care unit, complicating pain assessment due to mixed age groups. To address this, it is essential to use pain scales that are applicable to a wide age range. The Critical-Care Pain Observation Tool (CPOT) was developed to assess pain in both intubated and non-intubated adult patients. However, its applicability in pediatric patients has not been confirmed. The purpose of this study was to evaluate CPOT for critically ill pediatric patients.

**Methods:**

We conducted a prospective observational study in an eight-bed open PICU from January 2022 to March 2023. Three research nurses independently assessed pain using CPOT, the Face, Legs, Activity, Cry, Consolability (FLACC) scale, and an Observational Visual Analog Scale (VAS obs). Criterion-related and construct validity were examined using Spearman’s rank correlation coefficients between CPOT, VAS obs, and FLACC. Diagnostic performance was evaluated via ROC analysis using a FLACC score ≥ 4 as the reference. CPOT scores with and without medical interventions were compared using the Mann–Whitney U test, and inter-rater reliability was assessed with Cohen’s weighted κ.

**Results:**

Ninety-one patients were observed 165 times. CPOT strongly correlated with VAS obs (Spearman’s ρ =  0.87, p <  0.01) and FLACC (Spearman’s ρ =  0.84, p <  0.01). At a CPOT cut-off score of 3, sensitivity was 100% and specificity was 96.7%. CPOT effectively reflected pain levels during medical interventions (p <  0.01), and inter-rater reliability was high (weighted κ =  0.89, 95% CI: 0.799–0.941).

**Conclusions:**

This study suggests that CPOT may be a useful tool for pain assessment in pediatric patients.

## Introduction

Managing pain in pediatric patients in the Pediatric Intensive Care Unit (PICU) presents complex challenges that are influenced by various factors. Critically ill children in the PICU frequently undergo invasive procedures that are inherently painful, necessitating meticulous pain management approaches [1]. One of the primary challenges is the accurate assessment of pain, which is particularly problematic in critical care environments [2]. Various assessment tools and scales have been validated to evaluate pain levels in critically ill pediatric patients, including the Face, Legs, Activity, Cry, Consolability (FLACC) scale and COMFORT-B [3,4]. The indications for these tools for pediatric patients are also recommended in the guidelines [5].

However, it is important to note that not all critically ill pediatric patients are treated in dedicated PICUs, and in some facilities—both in Japan and elsewhere—pediatric and adult patients may share the same unit [6–8]. Caring for critically ill pediatric patients requires specialized skills; however, pediatric patients with critical conditions are not always managed exclusively in a PICU. In these mixed-age units, the requirement to appropriately assess patients of all ages can increase the workload of medical staff and potentially lead to inadequate pain assessment. Age-inclusive pain scales are essential for effective management. The Behavioral Pain Scale (BPS) has been evaluated in pediatric patients [9]. However, as this tool is primarily designed for intubated populations, its use in non-intubated children is limited, highlighting the need for more versatile pain assessment tools.

The Critical-Care Pain Observation Tool (CPOT) was originally validated in adult patients [10]. The tool evaluates four specific criteria that could conceivably be applicable to pediatric patients: facial expression, body movements, compliance with the ventilator for intubated patients, and vocalization for extubated patients. Despite its widespread use in adult ICUs, its applicability in critically ill pediatric patients remains underexplored [11].

This study aimed to evaluate the validity and reliability of the Critical Care Pain Observation Tool (CPOT) in pediatric patients in a critical care setting.

## Methods

### Study design and patients

This study was a prospective observational research project conducted between January 2022 and March 2023. The subjects were pediatric patients admitted to the eight-bed open PICU at the University of Tsukuba Hospital. Patients with central nervous system diseases, a history of neurological conditions such as hypoxia and cerebral palsy, and those receiving muscle relaxants were excluded from this study because of the potential difficulty in accurately assessing pain levels. Demographic and clinical data were collected from participants. A detailed explanation of the study was provided both verbally and in writing. As all participants in this study were minors, written informed consent was obtained from their parents or guardians. This consent process was documented, and written records were retained by the research team. In accordance with the ethical standards of the responsible committee on human experimentation (institutional or regional) and the 2013 version of the Helsinki Declaration, this study was approved by the Ethics Committee of the University of Tsukuba Hospital on August 8, 2016 (approval #H28-085, study title: The Study on Delirium, Pain, Sedation, and Withdrawal Symptoms in Pediatric Intensive Care Unit). As additional tools, including the CPOT, were incorporated into the study, amendments were submitted to the ethics committee and subsequently approved. All procedures performed in this study involving human participants complied with the ethical standards of the institution.

### Procedure

To assess the validity and reliability of the CPOT in measuring children’s pain, a team comprising three research nurses independently, blindly, and simultaneously employed three different pain measurement scales: CPOT, FLACC scale, and the observational Visual Analog Scale (VAS obs). To minimize potential bias, the VAS obs was administered prior to the CPOT and FLACC assessments. Multiple raters were employed to enhance inter-rater reliability testing.

### Pain scales

The CPOT was designed to assess pain in critically ill adult patients who are unable to communicate verbally due to conditions such as sedation, intubation, or their underlying diagnosis [10]. CPOT consists of four distinct categories: facial expression, body movements, compliance with the ventilator (intubated patients) or vocalization (extubated patients), and muscle tension—each rated on a three-point scale (0–2), with a total score of 0 to 8. A back-translated Japanese version of the CPOT was used in this study [12]. The FLACC scale is a behavioral assessment tool designed to quantify postoperative pain intensity in children [4]. Each of its five categories—face, legs, activity, cry, and consolability—is rated on a scale from 0 to 2, resulting in a total score ranging from 0 (no pain) to 10 (severe pain), with a threshold value of 4. This scale is also recommended by guidelines as a pain assessment tool for critically ill patients [5]. Our previous research has confirmed the validity and reliability of the Japanese version of the FLACC scale [13]. The FLACC scale is the approved tool currently employed to evaluate pain in the PICU of our hospital. The visual analog scale (VAS), first introduced in 1921, was a unidimensional tool for quantifying subjective pain intensity using a 10 cm line labeled from “no pain” to “worst imaginable pain” [14]. However, since younger pediatric patients cannot self-report their pain, we utilized the observational VAS (VAS obs) instead. The VAS obs is a unidimensional tool in which healthcare professionals assess and pain based on observed patient symptoms, using a 10 cm line ranging from ‘no pain’ to ‘worst imaginable pain’ similar to the VAS. The use of VAS obs for assessing the validity of pain scales in neonates and children has been reported in previous studies [15,16].

### Sample size

The sample size was calculated using the Spearman’s correlation coefficient. Because the required number of participants decreases as Spearman’s ρ increases (e.g., 0.7 =  18, 0.6 =  24, 0.5 =  33, 0.4 =  51), we chose ρ =  0.3 as the minimal threshold for detecting a “low” correlation. According to previous definitions, |ρ| <  0.3 indicates a negligible correlation, whereas 0.3 ≤  |ρ| <  0.5 represents a low correlation [17]. We aimed to detect this threshold at a significance level (α) of 0.05 and a power (1−β) of 0.8. From these calculations, we determined that we needed at least 89 participants.

### Statistical analysis

Patient characteristics, including demographics such as age, sex, mortality risk, intubation status, and diagnoses, were obtained from clinical charts. Criterion-related validity of the CPOT was assessed through its correlation with the VAS obs, while construct validity was evaluated via its correlation with the FLACC scale, using Spearman’s rank correlation coefficient (ρ). Receiver Operating Characteristic (ROC) curve analysis was used to determine the diagnostic performance of the CPOT, with an FLACC score of 4 or higher as the threshold. The Mann–Whitney U-test was used to compare differences in CPOT scores during medical interventions, such as intravenous catheterization, suctioning, and bathing. The inter-rater reliability for CPOT scores was assessed using Cohen’s weighted κ test.

Data analysis was conducted using Python programming language (version 3.8) and EZR (Saitama Medical Center, Jichi Medical University, Saitama, Japan), a graphical user interface for R (The R Foundation for Statistical Computing, Vienna, Austria) [18]. All statistical tests were two-sided, and a p-value of less than 0.05 was considered statistically significant. Bootstrap methods with 1,000 resamples were used to calculate 95% confidence intervals (CIs). Data manipulation was performed using the pandas library (version 1.2.3), and statistical computations were executed using the scikit-learn library (version 0.24.1).

## Results

In total, 135 patients were enrolled in this study. After excluding 20 patients with central nervous system diseases and an additional 24 patients with a history of neurological conditions, 91 pediatric patients were included in the study (Table 1). As shown in Table 2, these 91 patients were observed a total of 165 times (each subject was evaluated between one and five times). The median age of the included patients was 13 months (range: 0–214 months).

**Table 1:** Characteristics and diagnosis of study subjects.

Characteristics	No	Median (IQR) ± SD or %
Age in month	91	13 (2 - 45)
< 12 months	44	48.4
1–2 years	24	26.4
3–5 years	4	4.4
6–12 years	12	13.4
13–18 years	7	7.6
Sex, frequency		
Female	29	31.9
Male	62	68.1
Pediatric Risk of Mortality III	91	15 (10- 19.5)
Mechanical ventilation	59	64.8
Primary diagnoses		
Cardiac surgery	63	69.2
Respiratory insufficiency	13	14.3
Abdominal Surgery	6	6.6
Hematologic/oncologic	3	3.3
Other	6	6.6

**Table 2:** Characteristics and diagnosis of assessments.

Characteristics	No	Median (IQR) or %
Age in month	165	6 (1 - 27)
≦ 12 months	98	59.4
1–2 years	35	21.2
3–5 years	6	3.6
6–12 years	14	8.5
13–18 years	12	7.3
Sex, frequency		
Female	45	27.3
Male	120	72.7
Pediatric Risk of Mortality III	91	15 (11 - 25)
Mechanical ventilation	76	46
Sedatives and analgesics		
Use of midazolam	68	39.4
Use of Dexmedetomidine	86	52.1
Use of fentanyl	79	47.9
Primary diagnoses		
Cardiac surgery	126	76.4
Respiratory insufficiency	15	9.1
Abdominal Surgery	6	3.6
Hematologic/oncologic	3	1.8
Other	15	9.1

### Validity

As demonstrated in Table 3, criterion-related validity was assessed using CPOT and VAS obs scores, while construct validity was examined using Spearman’s rank correlation coefficient (ρ) between CPOT and FLACC. The correlation coefficients were high, with all values exceeding 0.7, indicating a strong and significant relationship (p <  0.01). This finding underscores the robust validity of CPOT in diverse patient groups.

**Table 3:** Spearman rho correlation between CPOT and VAS obs and between CPOT and FLACC.

			VAS obs	FLACC	*p*
		CPOT			
All observations	(n = 165)		0.87	0.84	< 0.01
No mechanical ventilation	(n = 89)		0.85	0.87	< 0.01
Mechanical ventilation	(n = 76)		0.91	0.80	< 0.01
< 12 months	(n = 98)		0.92	0.89	< 0.01
1 - 2 years	(n = 35)		0.83	0.74	< 0.01
3 - 5 years	(n = 6)		0.98	1.0	< 0.01
6 – 12 years	(n = 14)		0.86	0.88	< 0.01
13 – 18 years	(n = 12)		0.87	0.96	< 0.01

CPOT = The Critical-Care Pain Observation Tool; VAS obs = Observational Visual Analog Scale; FLACC = The Face, Legs, Activity, Cry = Consolability.

**Fig 1:**
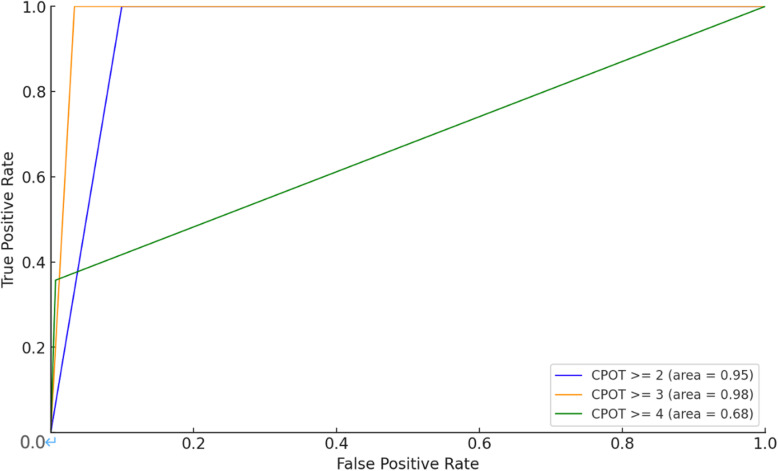
Receiver Operating Characteristic (ROC) curves for the Critical-Care Pain Observation Tool (CPOT) in critically ill pediatric patients, with different CPOT cut-off scores compared against the FLACC scale score of 4 or higher as the pain criterion. The blue line represents CPOT ≥  2 (AUC =  0.95), the yellow line represents CPOT ≥  3 (AUC =  0.98), and the green line represents CPOT ≥  4 (AUC =  0.89). The optimal CPOT cut-off score was identified as 3, with a sensitivity of 100% and a specificity of 96.7%.

The optimal cut-off score for CPOT was greater than 3, as demonstrated by the Receiver Operating Characteristic (ROC) curve (Fig 1). The AUC (Area Under the Curve), sensitivity, specificity, PPV (Positive Predictive Value), and NPV (Negative Predictive Value) at cut-off scores of ≥ 2, ≥ 3, and ≥ 4 are shown in Table 4. In particular, the CPOT cut-off of ≥ 3 yielded excellent diagnostic performance, with an AUC of 0.98, sensitivity of 100%, and specificity of 96.7%. The positive likelihood ratio was 30.2 and the negative likelihood ratio was less than 0.01 at this threshold, highlighting the clinical significance of this cut-off score. CPOT scores were significantly higher in patients who were undergoing medical interventions than in those who were not (p <  0.01).

**Table 4:** The AUC, sensitivity, specificity, positive likelihood ratio, and negative likelihood ratio for each CPOT score were calculated based on a FLACC score of 4 or higher.

CPOT Score	AUC	Sensitivity	Specificity	PPV	NPV
2 ≧	0.95	1	0.90	0.48	1
3 ≧	0.98	1	0.97	0.74	1
4 ≧	0.67	0.357	0.99	0.83	0.94

AUC = Area Under the Curve; PPV = Positive Predictive Value; NPV = Negative Predictive Value.

### Reliability

The inter-rater reliability of the CPOT scores across observers, as assessed by two nurses, was greater than 0.8, as demonstrated in Table 5, indicating strong agreement between nurses in evaluating pain using the CPOT scale. Weighted κ values ranged from 0.74 to 1.0 across different patient subgroups, further confirming the reliability of CPOT in this pediatric critical care setting.

**Table 5:** Measures of agreement between the observers.

Scale	κ	95%CI		*p*
All observations (n = 165)		Lower	Upper	
CPOT total score	0.89	0.8	0.94	<0.01
Facial expression	0.86	0.74	0.94	<0.01
Body movement	0.74	0.59	0.84	<0.01
Muscle tension	0.84	0.69	0.94	<0.01
Vent or Vocal	0.80	0.59	0.92	<0.01
No mechanical ventilation (n = 89)				
CPOT total score	0.88	0.76	0.95	<0.01
Mechanical ventilation (n = 76)				
CPOT total score	0.89	0.74	0.96	<0.01
< 12 months (n = 98)				
CPOT total score	0.86	0.59	0.94	<0.01
1 - 2 years (n = 35)				
CPOT total score	0.93	0.77	0.98	<0.01
3 - 5 years (n = 6)				
CPOT total score	1.0	NaN	NaN	<0.01
6 - 12 years (n = 14)				
CPOT total score	0.88	0.65	1.0	<0.01
13 - 18 years (n = 12)				
CPOT total score	0.75	0.31	0.94	<0.01

CPOT, The Critical-Care Pain Observation Tool; Vent, Ventilator (intubated patients); Vocal, Vocalization (extubated patients).

## Discussion

We evaluated the validity and reliability of the CPOT in critically ill pediatric patients. CPOT was originally developed to measure pain in critically ill adult patients who were unable to communicate verbally because of their diagnosis, sedation, or intubation [10]. Given its widespread international adoption and high regard for its validity and reliability in adult patient populations [11], we investigated whether this tool, initially conceived for adults, could also be applied in pediatric settings. The results of our study indicate that CPOT items were observable in pediatric patients, providing new insights its potential application and suggesting that it can be adapted effectively to younger populations.

In this study, CPOT scores exhibited a high correlation with VAS obs scores, as evaluated by the attending nurses. The VAS obs is a unidimensional tool designed to quantify pain intensity, utilizing a 10 cm line with endpoints labeled ‘no pain’ and ‘worst imaginable pain.’ This scale is widely used to compare pain between patients and to monitor the course of pain in individual patients. Previous research has employed the VAS obs to evaluate the validity of pain scales in pediatric populations [19,20]. Additionally, CPOT scores were highly correlated with the FLACC scale. This is particularly significant because the FLACC scale is frequently used to assess critically ill pediatric patients [21]. Our research team has extensive experience with the FLACC scale, which evaluates its domestic version [13]. CPOT demonstrated a high correlation with both the VAS obs and FLACC scales in a pediatric setting. Moreover, the strong correlation and the significant difference in CPOT scores during medical interventions collectively underline the scale’s capacity to capture fluctuations in pediatric pain states.

In addition, this study found that when using the FLACC scale as a benchmark for pain assessment, a CPOT positive threshold score of three or higher yielded the highest AUC. This differs from the original CPOT, which typically uses a cut-off score of 2 (ranging from 0 to 8 across four categories). However, it is important to note that the cutoff value may vary depending on the population and situation, and past reports have shown cutoff values ranging from 2 to 3 in adults [22]. This suggests that there is a high likelihood of multiple cut-off values being reported for pediatric populations in future studies.

The higher the value of weighted kappa, the stronger the agreement, with values ranging from 0 to 1. Values between 0.81 and 1.00 indicate “almost perfect” agreement, whereas those between 0.61 and 0.80 are considered “substantial” [23]. In our study, weighted kappa values for CPOT—including all observations and subgroups—fell within these ranges, demonstrating high levels of inter-rater reliability. The four dimensions of the CPOT demonstrated moderate to excellent inter-rater reliability. However, for ‘body movement’ the agreement was only ‘substantial’ likely because of differing assessments of subtle movements in children. Compared with adults, children tend to move more restlessly, and this characteristic may have impacted the consistency of the evaluations. Therefore, special attention may be needed when assessing ‘body movement’ in pediatric patients. In prior research, the agreement among nurses for scoring CPOT has also been reported to be ‘moderate’ [24,25]. Considering these results, the inter-rater reliability obtained in our study could indicate the reliability of CPOT in pediatric patients.

Pain assessment is limited in some areas [26–28], and we believe that one of the reasons is the inconvenience of switching between assessment tools depending on each patient’s age. Implementing CPOT in mixed-age intensive care units could address this issue by allowing both pediatric and adult patients to be evaluated with a single tool, thereby promoting consistency in pain assessment across different age groups. Furthermore, CPOT’s applicability to both intubated and extubated patients simplifies the measurement process, making it feasible to monitor pain continuously throughout the course of critical care. Such a unified approach may not only streamline clinical workflows but also foster more integrated pain management protocols. Taken together, these advantages underscore the value of exploring CPOT’s broader adoption in settings where patient demographics and ventilation status frequently vary.

This study had several limitations. First, patients with neurological conditions were excluded. Previous studies have used the FLACC and CPOT scales to assess these patient populations [29,30]. The evaluation of pain in this group is very important, and it is necessary to investigate whether CPOT can be used for assessment in the future. We are considering an evaluation of this group in future research. Excluding these patients might introduce a selection bias, as neurological impairment can significantly alter pain perception and expression. Second, the assessment data were analyzed only by a single pair of nurses. Therefore, it should be considered that this pair could have unconsciously assigned favorable VAS scores to the still unestablished CPOT and FLACC scales. In future studies, it will be important to include a larger number of nurses to confirm continued inter-rater reliability. Third, this study was conducted as an initial validation test in a single ICU with a relatively small number of pediatric patients and limited associated clinical diagnoses. Future trials involving a larger cohort, multiple PICU, and a wider spectrum of primary clinical diagnoses (e.g., surgeries other than cardiovascular, primary pulmonary disease, acute infection) are required for further validation and foundational data collection. To address these limitations, we recommend a multi-center approach to enhance generalizability, alongside refining the scoring criteria to better account for varied neurological and developmental statuses. Moreover, it is crucial to validate CPOT among patients receiving high flow nasal cannula oxygen and non-invasive positive ventilation, as these methods of respiratory support are increasingly being utilized on a consistent basis.

Overall, this study contributes to the existing literature by offering an initial demonstration that CPOT is both valid and reliable in critically ill pediatric populations, thereby expanding its known scope from adult-only usage. While our findings highlight CPOT’s feasibility and potential to unify pain assessment across age groups, future research will be critical for corroborating and refining these observations through larger and more diverse patient populations.

## Conclusions

The results of this study suggest that the CPOT could be a useful tool for pain assessment in pediatric patients. Further studies are urgently required to validate these findings.

## Supporting information

S1 File Assesment. (XLSX)

S2 File Patient. (XLSX)
